# Supplementary material for: Strong Substrate Strain Effects in Multilayered WS2 Revealed by High-Pressure Optical Measurements
Source: ACS Appl Mater Interfaces. 2022 Apr 20;14(17):19857–68. doi: 10.1021/acsami.2c01726 (PMC9073841; doi:10.1021/acsami.2c01726)
Supplement: Supplementary file 1 — am2c01726_si_001.pdf [file am2c01726_si_001.pdf]

**Supporting Information: Strong substrate strain effects in multilayered $WS_2$ revealed by high-pressure optical measurements**

Robert Oliva,[1*] Tomasz Wozniak,[1] Paulo E. Faria Jr.,[2] Filip Dybala,[1] Jan Kopaczek,[1] Jaroslav Fabian,[2] Paweł Scharoch,[1] Robert Kudrawiec[1]

[1] Department of Semiconductor Materials Engineering, Faculty of Fundamental Problems of Technology, Wroclaw University of Science and Technology, Wybrzeże Wyspiańskiego 27, 50-370 Wrocław, Poland

[2] Department of Physics, University of Regensburg, 93040 Regensburg, Germany.

[*] Corresponding author: robert.oliva.vidal@pwr.edu.pl

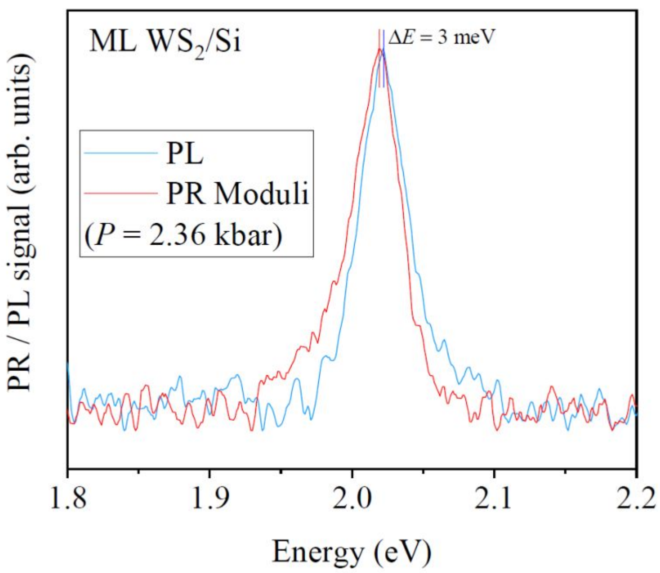


**Fig. S1.**

Moduli of photoreflectance and photoluminescence spectra of a $WS_2$ monolayer deposited on a Si substrate acquired at a pressure of 0.236 GPa. A very small Stokes shift (≈3 meV) can be observed.

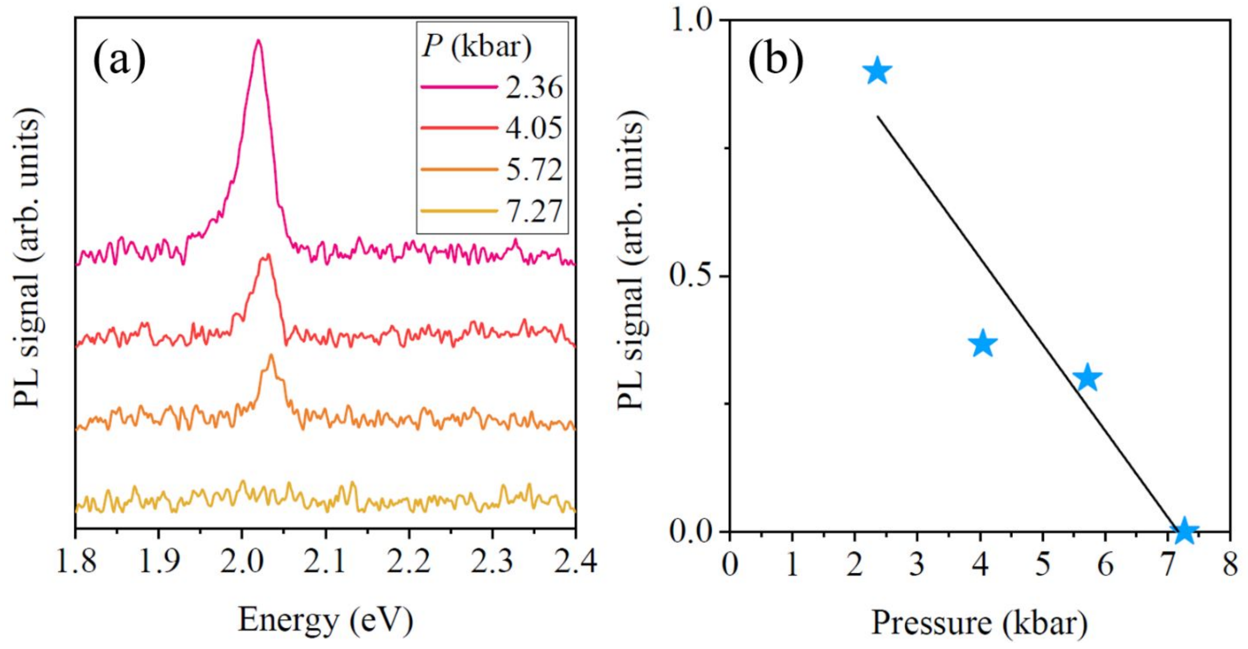


**Fig. S2.** (a) Photoluminescence spectra of a $WS_2$ monolayer deposited on Si acquired at different pressures, a smooth substrate has been subtracted for clarity. (b) The PL signal monotonically decrease with increasing pressure, vanishing at 0.72 GPa.

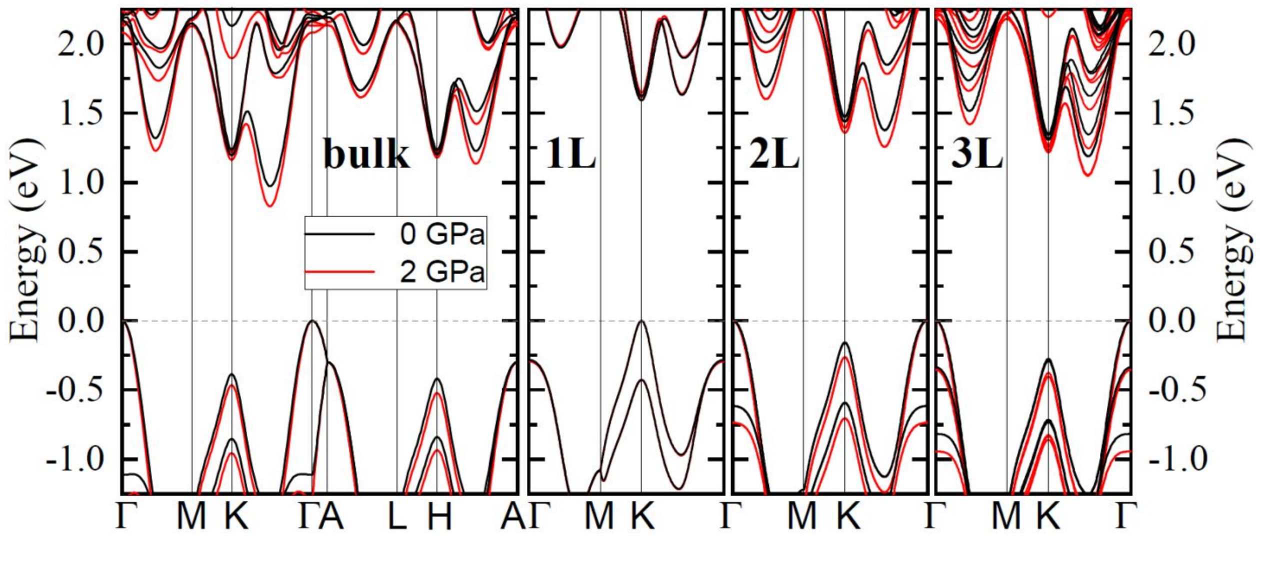


**Fig. S3.** Band structures of bulk as well as 1L, 2L and 3L $WS_2$ on a sapphire substrate at pressures of 0 and 2 GPa.

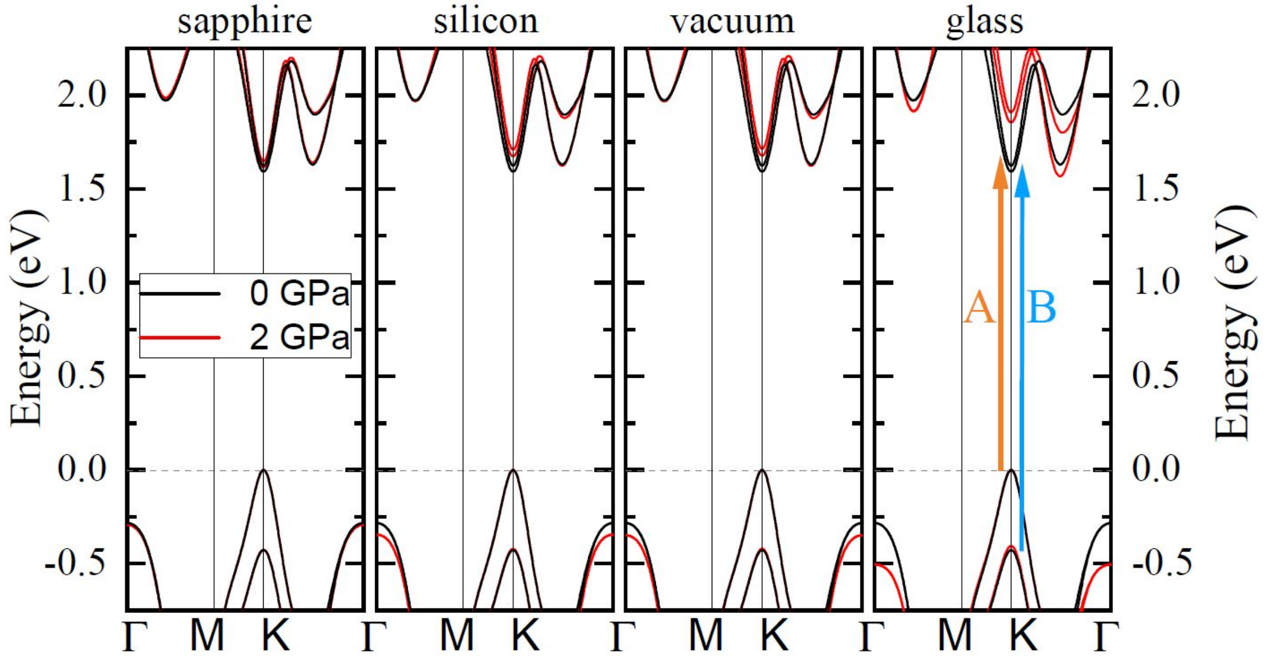


**Fig. S4.** Band structures of 1L $WS_2$ on different substrates at pressures of 0 and 2 GPa.

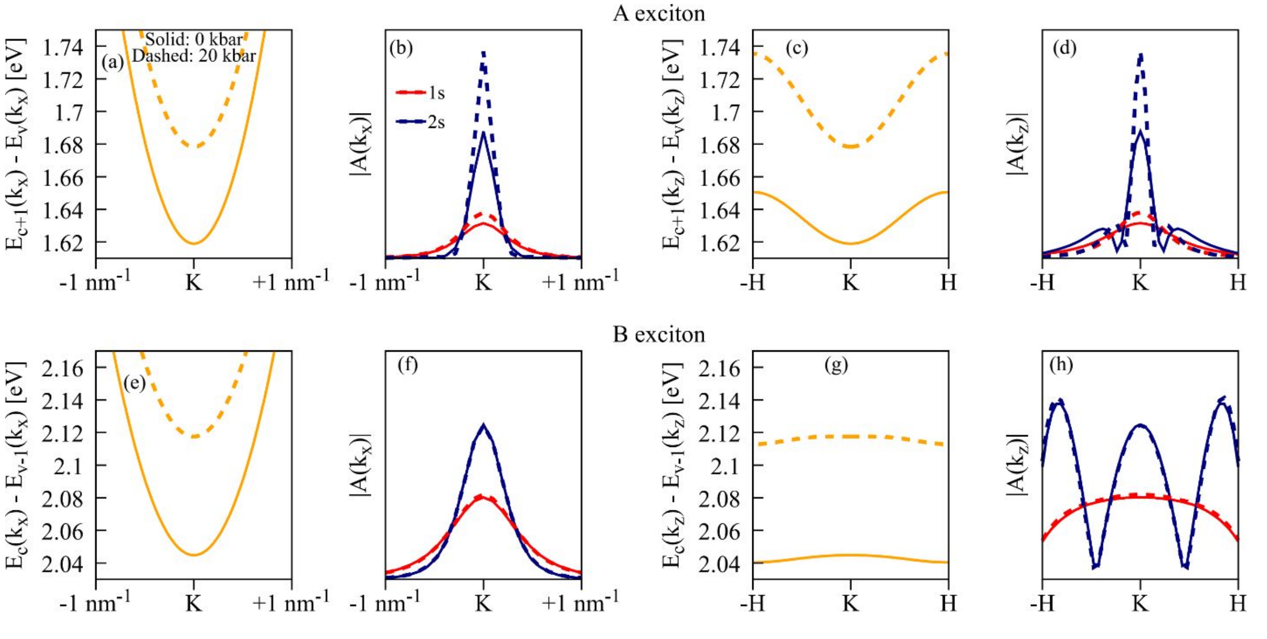


**Fig. S5. Top panel**: A exciton. **Bottom panel**: B exciton in bulk $WS_2$. **(a) and (e)**: Energy difference of conduction and valence bands in $k_Z$=0 plane and **(c), (g)** along the $k_Z$ direction. **(b) and (f)** Exciton wave functions of 1s (red) and 2s (blue) states in $k_Z$=0 plane and **(d), (h)** along $k_Z$ direction. 0 and 20 kbar pressures are represented by solid and dashed lines, respectively.

| P (kbar) | $m_{v-1}$ | $m_v$ | $m_c$ | $m_{c+1}$ | $\varepsilon_{xx}$ | $\varepsilon_{zz}$ |
|---|---|---|---|---|---|---|
| 0 | -0.48 | -0.35 | 0.36 | 0.27 | 14.084 | 5.987 |
| 20 | -0.48 | -0.36 | 0.37 | 0.28 | 14.470 | 6.879 |

**Table S-I.** Band effectives masses and static dielectric tensor components calculated for bulk $WS_2$ at 0 and 20 kbar.

| | | 1L | | | 2L | | 3L | |
|---|---|---|---|---|---|---|---|---|
| substrate | P (kbar) | $\mu_A$ | $\mu_B$ | $\varrho_0$ | $\mu_A$ | $\mu_B$ | $\mu_A$ | $\mu_B$ |
| freestanding | 0 | 0.150 | 0.207 | 39.74 | 0.151 | 0.208 | 0.152 | 0.209 |
| freestanding | 20 | 0.154 | 0.209 | 39.32 | | | | |
| sapphire | 20 | 0.149 | 0.206 | 39.55 | 0.152 | 0.207 | 0.153 | 0.209 |
| silicon | 20 | 0.154 | 0.209 | 39.34 | | | | |
| glass | 20 | 0.168 | 0.222 | 38.89 | | | | |

**Table S-II.** Calculated parameters for 1L, 2L and 3L $WS_2$ at 0 and 20 kbar, in which $\mu_A$ is the reduced mass for the X=A, B exciton and $\varrho_0$ is the screening length (2D polarizability).

| Reference | dE/dP (meV/GPa) | E (eV) | Comments |
|---|---|---|---|
| Dybała et al.[1] | 27 | 1.993 | PR, transition A. |
| Dybała et al.[1] | 41 | 2.432 | PR, transition B. |
| Dybała et al.[1] | -61.1 | 1.12 (approx.) | Calc, Indirect (?-G) |
| Shang et al.[2] | −28.4 | | Calc. indirect gap. (M?-G) |
| Shang et al .[2] | −16.5 | | Calc. direct gap at M (trans A) (structure 2Hc) |
| Shang et al .[2] | 23 | | Calc. direct gap at H |
| Shang et al .[2] | −20 | | Calc. direct gap at G |
| Shen et al [3] | −24 | | $WSe_2$, absorbance |

**Table S-III.** Reported pressure coefficients of excitonic transitions for selected bulk TMDCs.

| Reference | *dE/dP* (meV/GPa) | *E* (eV) | Comments |
|---|---|---|---|
| Han et al. [4] | 0.9 | | Trans. A. On Diamond. Abs. |
| Han et al. [4] | 0.7 | | Trans. B. On Diamond. Abs. |
| Han et al. [4] | 2.1-2.25 | | Trans A. On Si/SiO2, PL |
| Han et al. [4] | 0.53-1.24 | | Trans A. On Diamond, PL |
| Han et al. [4] | 1.04 | | Trion. On Si/SiO2, PL |
| Han et al. [4] | 0.3 | | Trion. On Diamond. PL |
| Han et al. | 0.54 | | Trans. A. On Diamond. Abs. |
| Kim et al. [5] | 3.35-5.4 | 1.946 | PL, on Si/SiO2 |
| Han et al. [4] | 0.31 $cm^{-1}$/GPa | | A1', on Diamond |
| Han et al. [4] | 0.39 $cm^{-1}$/GPa | | A1', on Si/SiO2 |
| Ye et al. [6] | 3.15 | | $WSe_2$. PL. Trans at K. |
| Ye et al. [6] | 2.7 | | $WSe_2$, Bilayer. PL. Trans at K. |
| Ye et al. [6] | −0.3 | | $WSe_2$, Indirect Λ–K, monolayer |
| Ye et al. [6] | −2.2 | | $WSe_2$, Indirect Λ–K, bilayer |

**Table S-IV.** Reported pressure coefficients of excitonic transitions for mono- and multilayers of $WS_2$ (unless otherwise stated in the comment column) deposited on different substrates.

| | $E_B$ at 0 kbar (meV) | $E_B$ at 20 kbar (meV) | $dE_B/dP$ (meV/GPa) |
|---|---|---|---|
| A1s | 43.3 (41.2) | 35.4 (33.1) | -4 |
| A2s | 14.8 | 10.4 | -2.2 |
| B1s | 63.4 | 57.7 | -2.9 |
| B2s | 30.8 | 29.1 | -0.9 |

**Table S-V.** Binding energies for A and B, 1s and 2s, excitons and their pressure coefficients in bulk $WS_2$ calculated from BSE. Values in parentheses are obtained from Gerlach-Pollmann model.

| | $dE_{QP}/dP$ | $dE_B/dP$ | $dE/dP$ | $dE/dP$ experiment |
|---|---|---|---|---|
| A | 29.7 | -4 | 33.7 | 24±1 |
| B | 36.4 | -2.9 | 39.3 | 36±3 |
| A* as A2s | 29.7 | -2.2 | 31.9 | 34±2 |
| A* as H-point | 42.2 | | | |

**Table S-VI.** Calculated pressure coefficients (in meV/GPa) of A, B and A* excitons in bulk $WS_2$ with band-to-band (from DFT) and excitonic (from BSE) contributions. Experimental values with uncertainties are included.

| | $dE_{QP}/dP$ | $dE_B/dP$ $\varepsilon(P)$ and $E(k,P)$ | $dE_B/dP$ $\varepsilon(P)$ | $dE/dP$ | $dE/dP$ experiment |
|---|---|---|---|---|---|
| freestanding | 45.5 | 3.8 | 3.8 | 41.7 | |
| sapphire | 12.1 | -0.1 | 1.3 | 12.2 – 10.8 | 11±1 |
| silicon | 43.2 | 0.9 | 0.9 | 42.3 | 30±4 |
| glass | 142.5 | 7.4 | 2.0 | 135.1 – 140.5 | 123±28 |

**Table S-VII.** Calculated pressure coefficients (in meV/GPa) of A exciton in 1L $WS_2$ with band-to-band (from DFT) and excitonic (from BSE) contributions neglecting the contribution from the pressure-dependence of the dielectric constant of the pressure transmitting media. Experimental values with uncertainties are included.

| | $dE_{QP}/dP$ | $dE_B/dP$ $\varepsilon(P)$ and $E(k,P)$ | $dE_B/dP$ $\varepsilon(P)$ | $dE/dP$ | $dE/dP$ experiment |
|---|---|---|---|---|---|
| freestanding | 45.5 | -27.58 | | 73.1 | |
| sapphire | 12.1 | -3.18 | -2.04 | 15.3 | 11±1 |
| silicon | 43.2 | -1.53 | -1.54 | 44.7 | 30±4 |
| glass | 142.5 | -3.07 | -8.29 | 145.6 | 123±28 |

**Table S-VIII.** Calculated pressure coefficients (in meV/GPa) of A exciton in 1L $WS_2$ with band-to-band (from DFT) and excitonic (from BSE) contributions including the contribution from the pressure-dependence of the dielectric constant of the pressure transmitting media, Daphne 7474. Experimental values with uncertainties are included.

## References


1. Dybała, F. *et al.* Pressure coefficients for direct optical transitions in $MoS_2$, $MoSe_2$, $WS_2$, and $WSe_2$ crystals and semiconductor to metal transitions. *Sci. Rep.* **6**, 26663 (2016).

2. Shang, J., Chen, P., Zhang, L., Fengxiao, Z. & Xuerui, C. The electronic and optical properties of Tungsten Disulfide under high pressure. *Chem. Phys. Lett.* **651**, 257–260 (2016).

3. Shen, P. *et al.* Linear Tunability of the Band Gap and Two-Dimensional (2D) to Three-Dimensional (3D) Isostructural Transition in WSe2 under High Pressure. *J Phys Chem C* **121**, 26019–26026 (2017).

4. Han, B. *et al.* Correlatively Dependent Lattice and Electronic Structural Evolutions in Compressed Monolayer Tungsten Disulfide. *J. Phys. Chem. Lett.* **8**, 941–947 (2017).

5. Kim, J.-S. *et al.* Towards band structure and band offset engineering of monolayer Mo (1− x ) W ( x ) S 2 via Strain. *2D Mater.* **5**, 015008 (2018).

6. Ye, Y. *et al.* Pressure-induced K–Λ crossing in monolayer WSe2. *Nanoscale* **8**, 10843–10848 (2016).
